# Association between 5p12 Genomic Markers and Breast Cancer Susceptibility: Evidence from 19 Case-Control Studies

**DOI:** 10.1371/journal.pone.0073611

**Published:** 2013-09-06

**Authors:** Xiaofeng Wang, Liang Zhang, Zixian Chen, Yushui Ma, Yuan Zhao, Abudouaini Rewuti, Feng Zhang, Da Fu, Yusong Han

**Affiliations:** 1 Department of Orthopedics Surgery, Zhongshan Hospital, Fudan University, Shanghai, People's Republic of China; 2 Department of Gastroenterology, Zhongshan Hospital, Fudan University, Shanghai, People's Republic of China; 3 Department of General Surgery, Zhongshan Hospital, Fudan University, Shanghai, People's Republic of China; Wake Forest University Health Sciences, United States of America

## Abstract

**Background:**

The association between polymorphisms on 5p12 and breast cancer (BC) has been widely evaluated since it was first identified through genome-wide association approach; however, the studies have yielded contradictory results. We sought to investigate this inconsistency by performing a comprehensive meta-analysis on two wildly studied polymorphisms (rs10941679 and rs4415084) on 5p12.

**Methods:**

Databases including Pubmed, EMBASE, Web of Science, EBSCO, and Cochrane Library databases were searched to find relevant studies. Odds ratios (ORs) with 95% confidence intervals (CIs) were used to assess the strength of association. The random-effects model was applied, addressing heterogeneity and publication bias.

**Results:**

A total of 19 articles involving 100,083 cases and 163,894 controls were included. An overall random-effects per-allele OR of 1.09 (95% CI: 1.06–1.12; *P = *4.5×10^−8^) and 1.09 (95% CI: 1.05–1.12; *P* = 4.2×10^−7^) was found for the rs10941679 and rs4415084 polymorphism respectively. Significant results were found in Asians and Caucasians when stratified by ethnicity; whereas no significant associations were found among Africans/African-Americans. Similar results were also observed using dominant or recessive genetic models. In addition, we find both rs4415084 and rs10941679 conferred significantly greater risks of ER-positive breast cancer than of ER-negative tumors.

**Conclusions:**

Our findings demonstrated that rs10941679-G allele and rs4415084-T allele might be risk-conferring factors for the development of breast cancer, especially in Caucasians and East-Asians.

## Introduction

Breast cancer (BC) is one of the most commonly occurring epithelial malignancies in women, with an estimated 1 million new cases and over 400,000 deaths annually worldwide [1]. Despite much investigation, the causes are not yet fully understood. Accumulated evidence suggests that multiple genetic and environmental factors, as well as the interplay between these factors, determine the phenotype. The well-known risk factors for BC include age at menarche, age at menopause, parity, age at first live birth, cigarette smoking, alcohol consumption, and hormone replacement therapy [2]. Genetic determinants including several high and moderate penetrance genes (BRCA1, BRCA2, BRIP1, CHEK2, PALB2, PTEN, and TP53) have been identified as BC susceptibility gene through the candidate gene approach in the past decade [3]. After accounting for all the known breast cancer loci, more than 75% of the familial risk of the disease remains unexplained [4].

Recently, a genome-wide association (GWA) study conducted in European ancestry (EA) population by Stacey et al. identified two genetic susceptibility loci, rs10941679 and rs4415084, at chromosome 5p12 were associated with BC risk [5]. Later, another study directed by Thomas et al. confirmed the single-nucleotide polymorphism (SNP) rs4415084 might affect BC risk in the EA population [6]. To date, the relationship between polymorphisms at 5p12 and BC or related phenotypes has been widely investigated. As stated by McClellan and King, many if not most of the genetic polymorphisms that are reported to be associated with common disorders in GWA studies are factually spurious associations caused by subtle differences in ancestry between the populations being studied (known as “cryptic population stratification”) [7]. Moreover, based on the fact that individual studies with insufficient sample sizes lack sufficient statistical power to detect the common variants with tiny effects on breast carcinogenesis, the results are not reproducible. To clarify this inconsistency and to establish a comprehensive picture of the relationship between 5p12 rs10941679 and rs4415084 polymorphism with BC risk, we therefore conducted a meta-analysis of all available published studies.

## Materials and Methods

### Literature search strategy

Papers published before the end of May 2013 were identified through a search of Pubmed, SCOPUS, ISI web of knowledge, Embase and Cochrane databases using the following terms: “breast cancer”, or “breast carcinoma”, and “chromosome 5p12”, or “rs10941679” or “rs4415084” and “polymorphism”, or “variation”. The titles and abstracts of potential articles were screened to determine their relevance, and any clearly irrelevant studies were excluded. The full texts of the remaining articles were read to determine whether they contained information on the topic of interest. All reference lists from the main reports and relevant reviews were hand searched for additional eligible studies not indexed by Medline.

### Eligible studies and data extraction

Eligible studies had to meet all of the following criteria: (1) original papers containing independent data which have been published in peer-reviewed journal, (2) genotype distribution information or odds ratio (OR) with its 95% confidence interval (CI) and *P*-value, (3) genotype distribution of control group must be consistent with Hardy–Weinberg equilibrium (HWE).

The following information was extracted independently and entered into separate databases by two authors from each qualified study: first author's surname, publication date, population ethnicity, source of control subjects, genotyping method, age, and the genotype counts in cases and controls. The results were compared, and disagreements were discussed among all authors and resolved with consensus. For studies including subjects of different ethnic groups, data were extracted separately. Meanwhile, different case–control groups in one study were considered as independent studies. For each study, we did not define a minimum number of patients for inclusion in our meta-analysis.

### Quality assessment: extended-quality score

For association studies with inconsistent results on the same polymorphisms, the methodological quality should be assessed by appropriate criteria to limit the risk of introducing bias into meta-analyses or systematic reviews. A procedure known as ‘extended-quality score’ has been developed to assess the quality of association studies. The procedure scores each paper categorizing it as having ‘high’, ‘median’ or ‘poor’ quality. Detailed procedure of the quality assessment was previously described [8].

### Statistical methods

The meta-analysis examined the association between each polymorphism and the risk of BC, for the: (i) allele contrast, (ii) recessive, and (iii) dominant models [9]. Crude ORs with 95% CIs were calculated using raw data, according to the method of Woolf B [10]. Cochran's Q-statistic test and *I^2^*-test were performed to assess possible heterogeneity in the combined studies [11], [12]. Both fixed-effects (Mantel–Haenszel method) [13] and random-effects (DerSimonian–Laird method) [14] models were performed to calculate the pooled ORs. Owing to a priori assumptions about the likelihood of heterogeneity between primary studies, the random-effects model, which usually is more conservative, was chosen. Sub-group analyses were performed by ethnicity, sample size (≤1000 cases and >1000 cases) and estrogen receptor (ER) status (ER-positive vs. ER-negative). Ethnic group was defined as Asians (e.g., Chinese, Japanese, and Korean), Caucasians (i.e. people of European origin) and Africans/African-Americans. One-way sensitivity analysis was performed to assess the stability of the results, namely, a single study in the meta-analysis was deleted each time to reflect the influence of the individual data set to the pooled OR. Funnel plots and the Egger's test were used to identify small studies effects (linear regression analysis) [15]. All P values are two-sided at the P = 0.05 level. All of the statistical tests used in this meta-analysis were performed by STATA version 10.0 (Stata Corporation, College Station, TX).

## Results

### Study Characteristics

Based on our search strategy, the primary screening produced 103 potentially relevant articles. A total of 19 studies published between 2008 and 2012 were included according to our inclusion criteria, involving 100,083 cases and 163,894 controls [5], [6], [16]–[32]. Study selection process was shown in Figure S1. For the rs10941679 polymorphism, 15 studies were available, including a total of 84,765 cases and 143,866 controls. For the rs4415084 polymorphism, 14 studies involved a total of 39,937 cases and 73,718 controls. Genotype frequencies in controls were consistent with HWE for all included studies. Of the cases, 12% were Asians, 84% were Caucasians, and 4% were of African origins. The extended-quality scores ranged from 5 to 8, and 6 studies were given median quality, whereas 13 were given high quality. No ‘poor quality’ study was found. The main characteristics of these studies are described in Table 1.

**Table 1:** Characteristics of studies included in a meta-analysis of the association between 5p12-rs10941679, 5p12-rs4415084 and BC.

Reference	Year	Country	Ethnicity	Polymorphism	Cases/controls	Matching criteria	Genotyping method	Quality score
Chan [16]	2012	China	Asian	rs10941679, rs4415084	1175/1499	NA	TaqMan	Median
Huo [17]	2012	Nigeria	African	rs10941679, rs4415084	1509/1383	Age and region	GoldenGate	Median
Sueta [18]	2012	Japan	Asian	rs10941679	697/1394	Age and menopausal status	TaqMan	Median
Liu [19]	2012	China	Asian	rs10941679, rs4415084	846/882	Age and region	TaqMan	High
Kim [20]	2012	Korea	Asian	rs4415084	2257/2052	Age and region	Microarray, TaqMan	High
Dai [21]	2012	China	Asian	rs10941679, rs4415084	1792/1867	Age and region	TaqMan	High
Campa [22]	2011	USA, Europe	Caucasian, Asian, African-American	rs10941679, rs4415084	6396/9225	Ethnicity and age	Taqman	High
Fletcher [23]	2011	UK	Caucasian	rs4415084	7643/7443	Age and postmenopausal hormone use	Microarray, GoldenGate	High
Li [24]	2011	Sweden, Finland	Caucasian	rs4415084	1557/4584	Age and region	Microarray	High
Milne [25]	2011	Canada, Australia, USA, Korea, China, Europe, Thailand	Caucasian, Asian	rs10941679	45377/74253	Age and region	TaqMan	High
Bhatti [26]	2010	USA	Caucasian	rs10941679, rs4415084	776/997	Age	TaqMan	Median
Zheng [27]	2010	China	Asian	rs10941679	3039/3082	Age	Microarray	High
Antoniou [28]	2010	European, America	Caucasian	rs10941679	8534/7011	Age and region	iPLEX	High
Wang [29]	2010	USA, Europe, Australia	Caucasian	rs10941679, rs4415084	3030/2427	NA	Microarray	High
Ruiz-Narvaez [30]	2010	USA	African-American	rs10941679, rs4415084	886/1089	Age and region	iPLEX	Median
Zheng [31]	2009	USA	African-American	rs10941679	810/1784	Age	Massarray	Median
Mcinerney [32]	2009	Ireland	Caucasian	rs4415084	882/997	Family history and region	KASPar	High
Thomas [6]	2009	USA, Europe	Caucasian	rs10941679, rs4415084	7849/9835	Ethnicity, age and region	Microarray, TaqMan	High
Stacey [5]	2008	Iceland, Sweden, Holland, Spain	Caucasian	rs10941679, rs4415084	5028/32090	Ethnicity and age	Microarray, Nanongen Centaurus assays	High

NA: not available.

### 5p12 rs10941679 polymorphism

**Figure 1:**
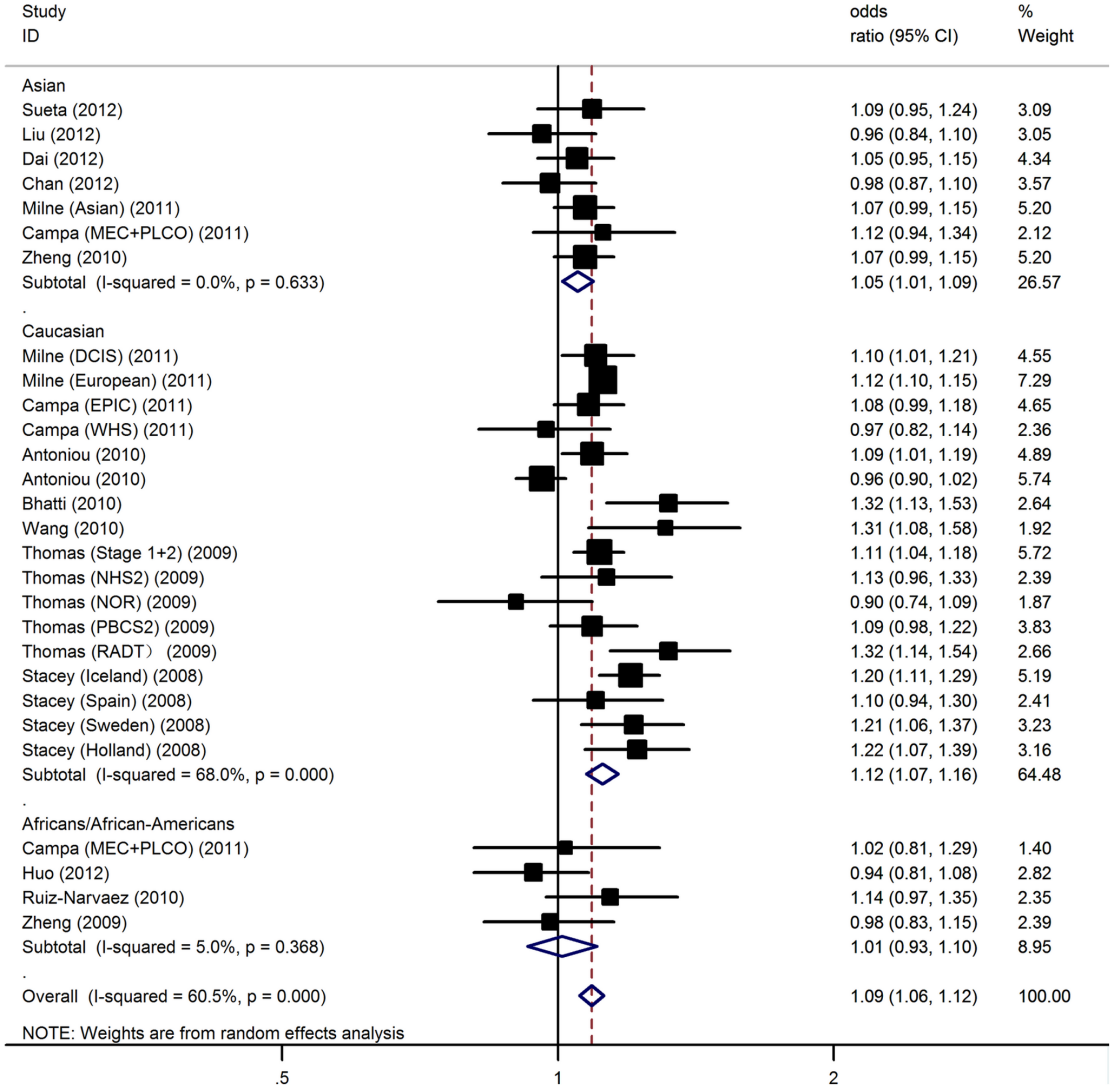
Forest plot for association of 5p12-rs10941679 polymorphism and BC risk.

In the overall analysis, the rs10941679 polymorphism was significantly associated with elevated BC risk with a per-allele OR of 1.09 [95% CI: 1.06–1.12, P(Z) = 4.5×10^−8^; Figure 1]. Significant associations were also found under dominant [OR = 1.11, 95% CI: 1.07–1.16, P(Z) = 6.9×10^−7^] and recessive [OR  = 1.13, 95%CI: 1.08–1.19, P(Z) = 5.1×10^−7^] genetic models.

In the stratified analysis by ethnicity, the OR was 1.05 [95% CI: 1.01–1.09, P(Z) = 0.01] in Asians and 1.12 [95% CI: 1.07–1.16, P(Z) = 3.8×10^−8^] in Caucasians. Among Africans/African-Americans, no significant association was found in all genetic models (Table 2). When stratifying for sample size, an OR of 1.11 [95% CI: 1.04–1.18, P(Z) = 0.008] and 1.08 [95% CI: 1.04–1.12, P(Z) = 3.3×10^−6^] resulted for G allele in small and larger studies, respectively. The data on genotypes of the polymorphism among cases and controls stratified by ER status were available in 7 studies involving 44,364 BC patients and 97,213 controls. Subsidiary analyses of ER status yielded a per-allele OR for ER-positive tumors of 1.16 [95% CI: 1.11–1.21, P(Z)  = 8.6×10^−11^], while no significant results were found ER-negative tumors [OR  =  1.02, 95% CI: 0.99–1.05, P(Z) = 0.21] (Figure S2).

**Table 2:** Results of meta-analysis for 5p12-rs10941679 polymorphism and BC risk.

Overall and subgroups analyses	No. of cases/ controls	G vs. A				Dominant Model				Recessive Model			
		OR (95%CI)	P(Z)	P(Q)	I^2^	OR (95%CI)	P(Z)	P(Q)	I^2^	OR (95%CI)	P(Z)	P(Q)	I^2^
Overall	84765/ 143866	1.09 (1.06–1.12)	4.5×10^−8^	3.2×10^−7^	60%	1.11 (1.07–1.16)	6.9×10^−7^	0.01	40%	1.13 (1.08–1.19)	5.1×10^−7^	7.2×10^−5^	56%
Ethnicity													
Asian	11093/ 11588	1.05 (1.01–1.09)	0.01	0.63	0%	1.07 (1.02–1.14)	0.02	0.37	0%	1.09 (1.04–1.20)	0.002	0.25	0%
Caucasian	70103/ 127620	1.12 (1.07–1.16)	3.8×10^−8^	2.4×10^−6^	68%	1.13 (1.08–1.18)	4.7×10^−6^	0.003	48%	1.14 (1.09–1.19)	4.0×10^−6^	0.01	43%
African/ African- Americans	3569/ 4658	1.01 (0.93–1.10)	0.81	0.37	5%	1.04 (0.97–1.13)	0.24	0.19	0%	1.28 (0.81–1.99)	0.36	0.09	3%
Sample size													
<1000	9650/ 15688	1.11 (1.04–1.18)	0.008	0.15	54%	1.13 (1.02–1.14)	0.01	0.10	38%	1.12 (1.09–1.17)	7.5×10^−5^	0.04	25%
≥1000	75115/ 128178	1.08 (1.04–1.12)	3.3×10^−6^	0.01	71%	1.11 (1.07–1.16)	2.5×10^−6^	0.06	46%	1.14 (1.08–1.20)	2.1×10^−6^	0.007	37%

### 5p12 rs4415084 polymorphism

**Figure 2:**
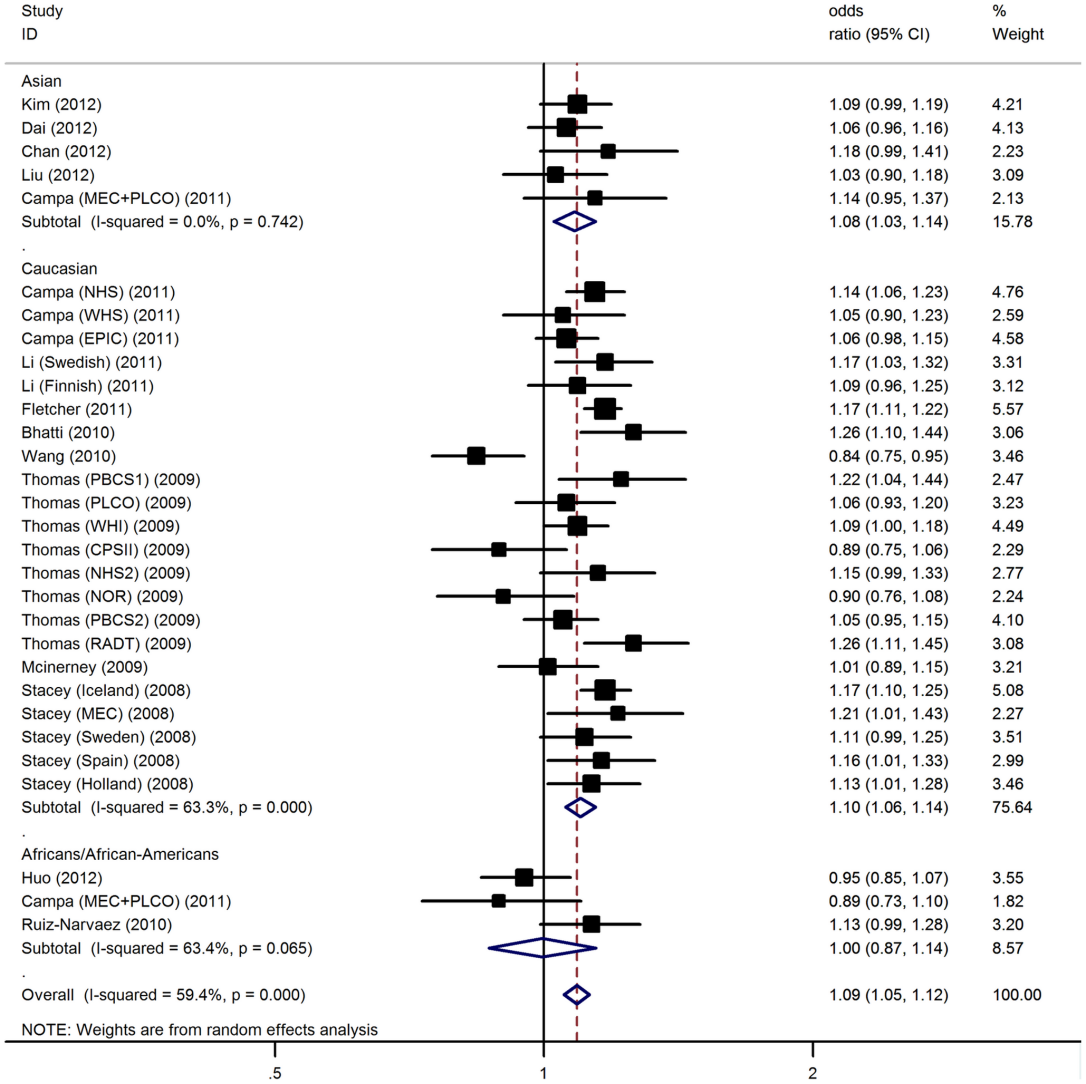
Forest plot for association of 5p12-rs4415084 polymorphism and BC risk.

The meta-analysis resulted in a statistically significant association between rs4415084 and BC. The overall OR for risk T allele was 1.09 [95% CI: 1.05–1.12, P(Z) = 4.2×10^−7^; Figure 2]. Significant results were also found under dominant [OR  = 1.10, 95% CI: 1.06–1.14, P(Z)  = 3.1×10^−7^] and recessive [OR  = 1.15, 95% CI: 1.09–1.21, P(Z)  = 6.8×10^−8^] genetic models (Table 3).

**Table 3:** Results of meta-analysis for 5p12-rs4415084 polymorphism and BC risk.

Overall and subgroups analyses	No. of cases/ controls	T vs. C				Dominant Model				Recessive Model			
		OR (95%CI)	P(Z)	P(Q)	I^2^	OR (95%CI)	P(Z)	P(Q)	I^2^	OR (95%CI)	P(Z)	P(Q)	I^2^
Overall	39937/ 73718	1.09 (1.05–1.12)	4.2×10^−7^	3.0×10^−8^	59%	1.10 (1.06–1.14)	3.1×10^−7^	3.9×10^−5^	46%	1.15 (1.09–1.21)	6.8×10^−8^	0.001	40%
Ethnicity													
Asian	6560/ 6805	1.08 (1.03–1.14)	0.004	0.74	0%	1.09 (1.02–1.13)	0.009	0.63	0%	1.12 (1.05–1.24)	0.01	0.32	0%
Caucasian	27906/ 59638	1.10 (1.06–1.14)	1.8×10^−6^	2.7×10^−5^	63%	1.12 (1.08–1.16)	4.7×10^−5^	2.0×10^−5^	37%	1.16 (1.11–1.20)	2.8×10^−7^	4.4×10^−5^	45%
African/ African- Americans	2790/ 2901	1.00 (0.87–1.14)	0.96	0.06	63%	1.05 (0.90–1.21)	0.55	0.08	21%	1.09 (0.91–1.30)	0.34	0.11	6%
Sample size													
<1000	13253/ 20627	1.10 (1.05–1.15)	1.4×10^−5^	0.03	42%	1.12 (1.07–1.19)	8.9×10^−5^	0.006	28%	1.18 (1.12–1.23)	7.2×10^−5^	2.3×10^−5^	47%
≥1000	26684/ 53046	1.07 (1.02–1.13)	0.005	9.3×10^−7^	75%	1.09 (1.04–1.14)	0.008	5.3×10^−5^	66%	1.14 (1.07–1.20)	0.001	5.6×10^−5^	58%

In the subgroup analyses by ethnicity, significant results were found in the Asians and Caucasians with a per-allele OR of 1.08 (95% CI: 1.03–1.14) and 1.10 (95% CI: 1.06–1.14), respectively. No significant associations were detected among Africans/African-Americans (OR = 1.00, 95% CI: 0.87–1.14). In considering sample size subgroups, the OR was 1.10 [95% CI: 1.05–1.15, P(Z) = 1.4×10^−5^] in small studies compared to 1.07 [95% CI: 1.02–1.13, P(Z) = 0.005] in large studies. The data on genotypes of the polymorphism among cases stratified by ER status were available in 6 studies including 20,000 cancer cases and 102,686 controls. The interaction between ER status and polymorphism, for which an OR of 1.14 [95% CI: 1.09–1.20, P(Z)  = 2.6×10^−7^] for ER-positive BC appeared in compared with individuals with the ER-negative BC [OR = 1.00, 95% CI: 0.95–1.06, P(Z)  = 0.96] (Figure S3).

### Sensitivity analyses and publication bias

In order to assess the stability of the results of the meta-analysis, we performed a sensitivity analysis through sequentially excluded individual studies. Statistically similar results were obtained after sequentially excluding each study, suggesting stability of the meta-analyses. Begg's funnel plot and Egger's test were performed to identify small studies effects. As shown in Figure S4 and S5, the shape of the funnel plots seemed symmetrical for both polymorphisms, suggesting no preferential publication of positive findings in smaller studies exists. The statistical results still did not show small study effects in these studies for rs10941679 (Egger's test, P = 0.59), and rs4415084 (Egger's test, P = 0.06).

## Discussion

Via a comprehensive meta-analysis, we evaluated the association of two common polymorphisms on 5q12 with the risk of BC. Overall results demonstrated that rs10941679-G allele and rs4415084-T allele might be risk-conferring factors for the development of BC in Caucasians and Asians, but not in Africans/African-Americans. Although potential sources of heterogeneity could not be easily eliminated, the present study, to our knowledge, is the first meta-analysis which involved a total of 100,083 cases and 163,894 from 19 studies to date dealing with the association of these two polymorphisms with BC susceptibility.

In meta-analysis, heterogeneity evaluation was always conducted in statistical analysis. Thus, several subgroup meta-analyses were performed according to ethnicity, sample size. In the stratified analysis by ethnicity, significant associations were found in Asians and Caucasians for the two polymorphisms in all genetic models; while no associations were detected among Africans. Such result could be due to the limited number of studies among Africans/African-Americans, which had insufficient statistical power to detect a slight effect or different linkage disequilibrium (LD) pattern of the two polymorphisms among Africans. Besides, the frequencies of the risk-association alleles in 5p12 are similar in European and Asian populations, but substantially lower in African descent [25], [29]–[31]. Furthermore, ER status may be particularly important given that some GWAS findings are specific to ER-positive and ER-negative cancers [5], [33] and because a higher proportion of African American are diagnosed with ER-negative cancers [34], [35], resulting in risk differences. Moreover, it is possible that variation at this locus has modest effects on BC, but environmental factors may predominate in its progress, and mask the effects of this variation. Specific environmental factors like lifestyle and diabetes that have been already well studied in recent decades [36]. It is still unknown whether the lifestyle characteristics of different populations influence the association between these polymorphisms and BC. The unconsidered factors mixed together may cover the role of the two polymorphisms in Africans/African-Americans. Meta-analysis is often dominated by a few large studies, which markedly reduces the evidence from smaller studies. By considering sample size, significantly increased BC susceptibility in 5p12 common variations was also found both in large and small studies for all genetic models. However, our results suggest an overestimation of the true genetic association by small studies.

ER status is known to affect prognosis of BC. Stratification of tumors by ER status indicated that the two SNPs (rs4415084 and rs10941679) on 5p12 confer risk, preferentially for ER-positive tumors, with no risk for ER-negative BC. Results from subgroup analyses on ER status of tumors were in agreement with previous reports [5], [25]. Findings from previous studies suggested that several SNPs are predominantly associated with ER-positive BC: 2q35-rs13387042 [22], TNRC9-rs3803662 [25], [37], 8q24-rs13281615 [38], FGFR2-rs2981582 [39]. The present findings support the notion that ER-positive and ER-negative tumors have different genetic components to their risks.

The biological mechanism through which genetic variations in 5p12 influences BC risk remains unclear. rs10941679 and rs4415084 are in a region of high LD as reported by Stacey et al [5]. MRPS30 (also known as PDCD9, programmed cell death protein 9) is the only RefSeq gene in this region. It is important to recognize that MRPS30, which is homologous to the pro-apoptotic p52 chicken gene [40], encodes a component of the small subunit of the mitochondrial ribosome, and is likely to be involved in pre-apoptotic events [41], [42]. In addition, some studies have showed that MRPS30 is not expressed in normal breast luminal epithelial cells, but it is up-regulated in infiltrating ductal carcinomas [43]. Moreover, it was also a part of a gene expression profile that differentiated ER-positive from ER-negative breast tumors [42]. As noted above, the associations observed in our study were stronger for ER-positive disease than for ER-negative disease. Interestingly, a SNP rs3761648 in 5′ near of MRPS30 is highly correlated with the SNP rs4415084 (r^2^ = 0.83 in HapMap CHB, r^2^ = 0.72 in HapMap CEU) and located at a site of H3K4Me3 histone modification marks. Therefore, it is possible that rs4415084 might be a proxy for other potentially functional SNPs, such as rs3761648, that influence MRPS30 expression and in consequence the cancer risk. Although the exact function of this gene is unknown, the apoptotic pathway is likely to be involved in mitigating DNA damage. Given the multifaceted and closely-linked nature of the DNA repair, cell-cycle and apoptotic pathways, it is likely that there are complex polygenic factors underlying the observed interactions of MRPS30 with BC [26].

The strengths of this study include the very large sample size, no deviation from HWE, and the high quality of the qualified studies. However, our current study should be interpreted with several technical limitations in mind. First, heterogeneity is a potential problem when interpreting all the results of meta-analysis. Although we minimized the likelihood by performing a careful search for published studies, using the explicit criteria for study inclusion, the significant between-study heterogeneity still existed in most of comparison. The presence of heterogeneity can result from differences in the age distribution, menopause status, environment factors, prevalence lifestyle factors and so on. Second, the vast majority of white subjects in the study are of European descent, and statistical power for analyses in other ethnicities is limited. Because the sample size was considerably smaller for Africans/African-Americans studies, the main conclusions from this manuscript are based on analyses among white European and Asian women. Future studies including larger numbers of Africans/African-Americans are necessary to clarify the consistency of findings across ethnic groups. Third, the subgroup meta-analyses considering interactions between common variations at 5p12 genotype and ER status with BC risk, were performed on the basis of a fraction of all the possible data to be pooled, so selection bias may have occurred and our results may be over inflated. In this context, more reliable results can be expected if individual data are available for a pooled analysis. Finally, lack of individual-level data prevent us from making an adjustment estimates to identify any interactions between genetic variation and clinical traits.

Taken together, we have expanded previously individual studies by providing the convincing evidence that MRPS30 gene rs10941679 and rs4415084 polymorphisms at 5p12 might be risk-conferring factors for the development of BC in Asians and Caucasians, but not in Africans/African-Americans. Further studies should investigate the markers on and adjacent to 5p12 to clarify whether the present association is causal or due to linkage disequilibrium.

## Supporting Information

Figure S1 Study selection process.
(TIF)

Figure S2 Per-allele ORs and 95% CIs for the association between 5p12-rs10941679 and breast cancer risk by ER status.
(TIF)

Figure S3 Per-allele ORs and 95% CIs for the association between 5p12-rs4415084 and breast cancer risk by ER status.
(TIF)

Figure S4 Begg's funnel plot of 5p12-rs10941679 polymorphism and BC risk.
(TIF)

Figure S5 Begg's funnel plot of 5p12-rs4415084 polymorphism and BC risk.
(TIF)

Checklist S1 (DOC)
