# Supplementary figures and images for: Association between 5p12 Genomic Markers and Breast Cancer Susceptibility: Evidence from 19 Case-Control Studies
Source: PLoS One. 2013 Sep 6;8(9):e73611. doi: 10.1371/journal.pone.0073611 (PMC3765311; doi:10.1371/journal.pone.0073611)

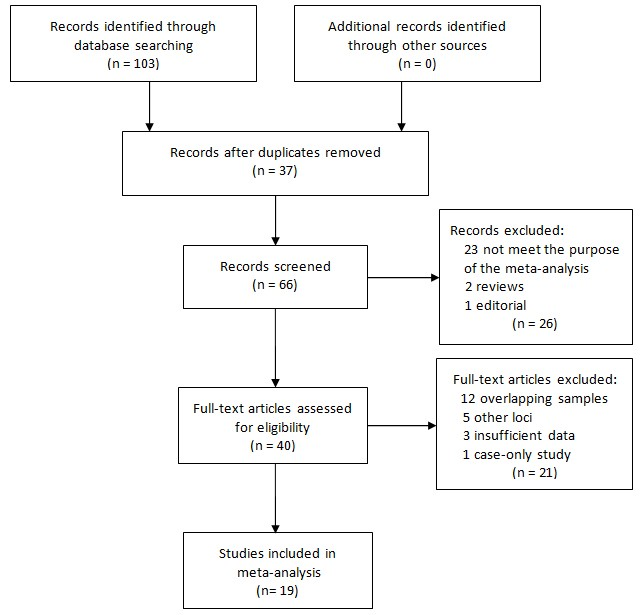

Supplement: Figure S1 — Study selection process. (TIF) [file pone.0073611.s001.tif]

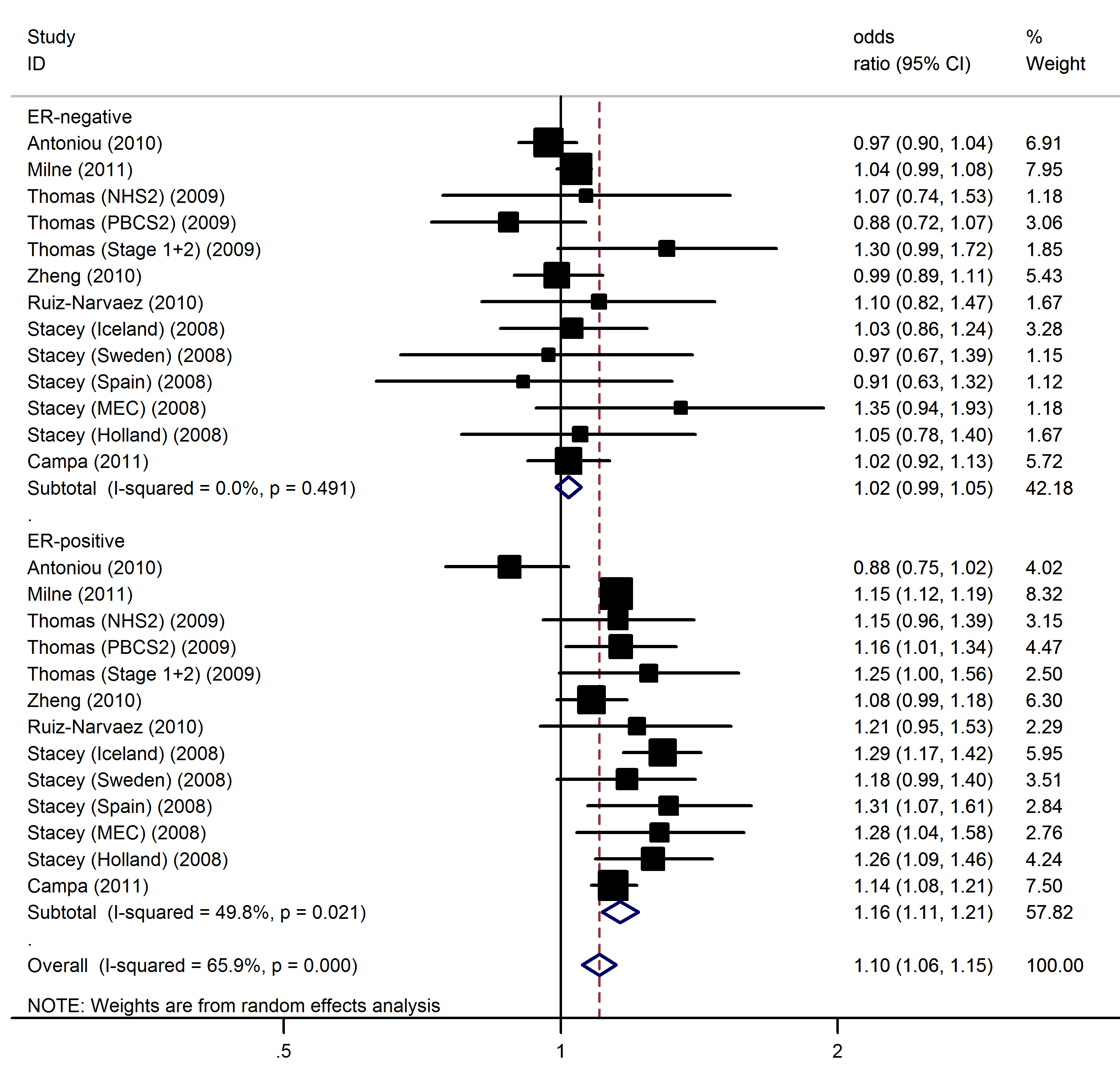

Supplement: Figure S2 — Per-allele ORs and 95% CIs for the association between 5p12-rs10941679 and breast cancer risk by ER status. (TIF) [file pone.0073611.s002.tif]

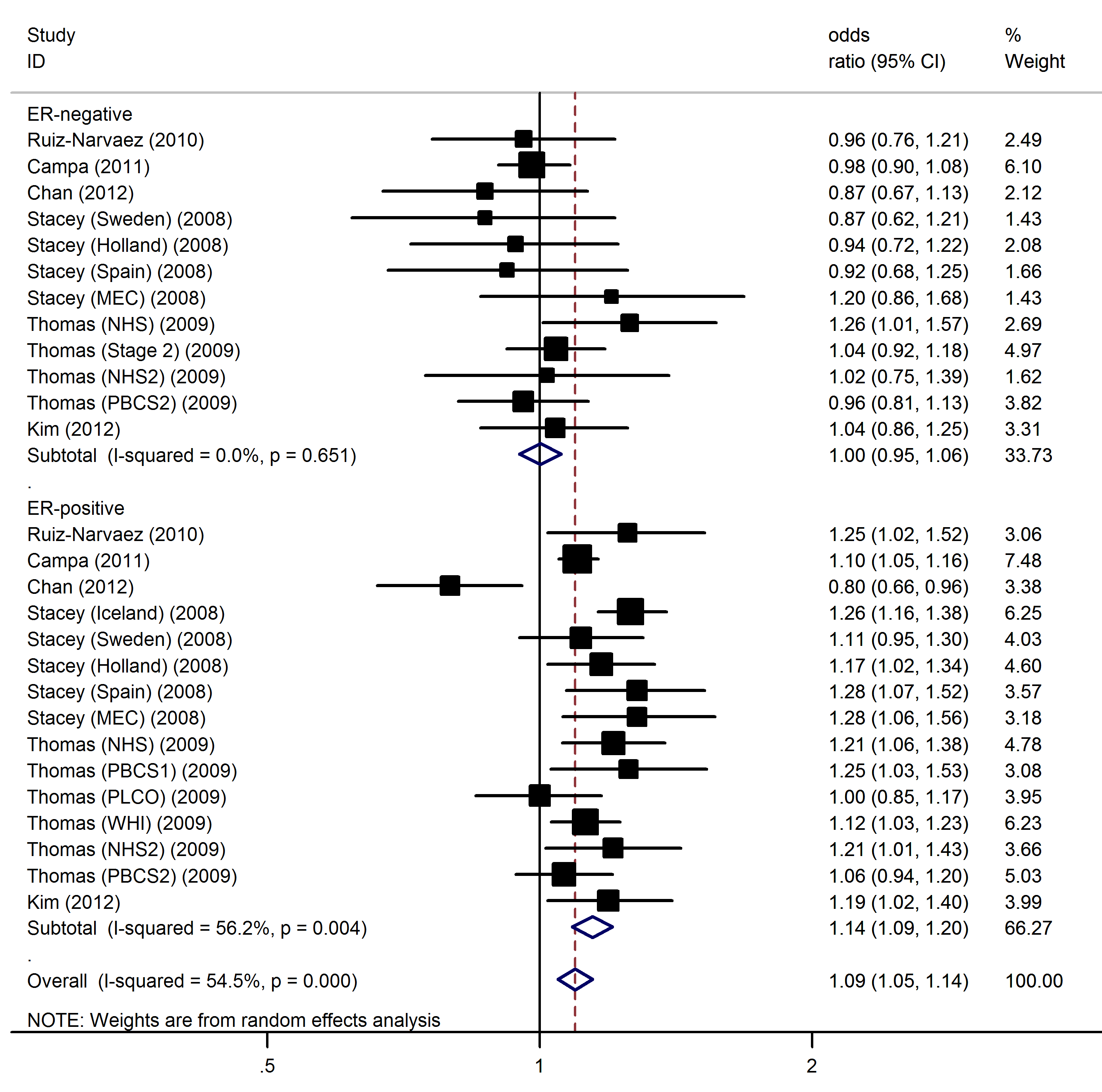

Supplement: Figure S3 — Per-allele ORs and 95% CIs for the association between 5p12-rs4415084 and breast cancer risk by ER status. (TIF) [file pone.0073611.s003.tif]

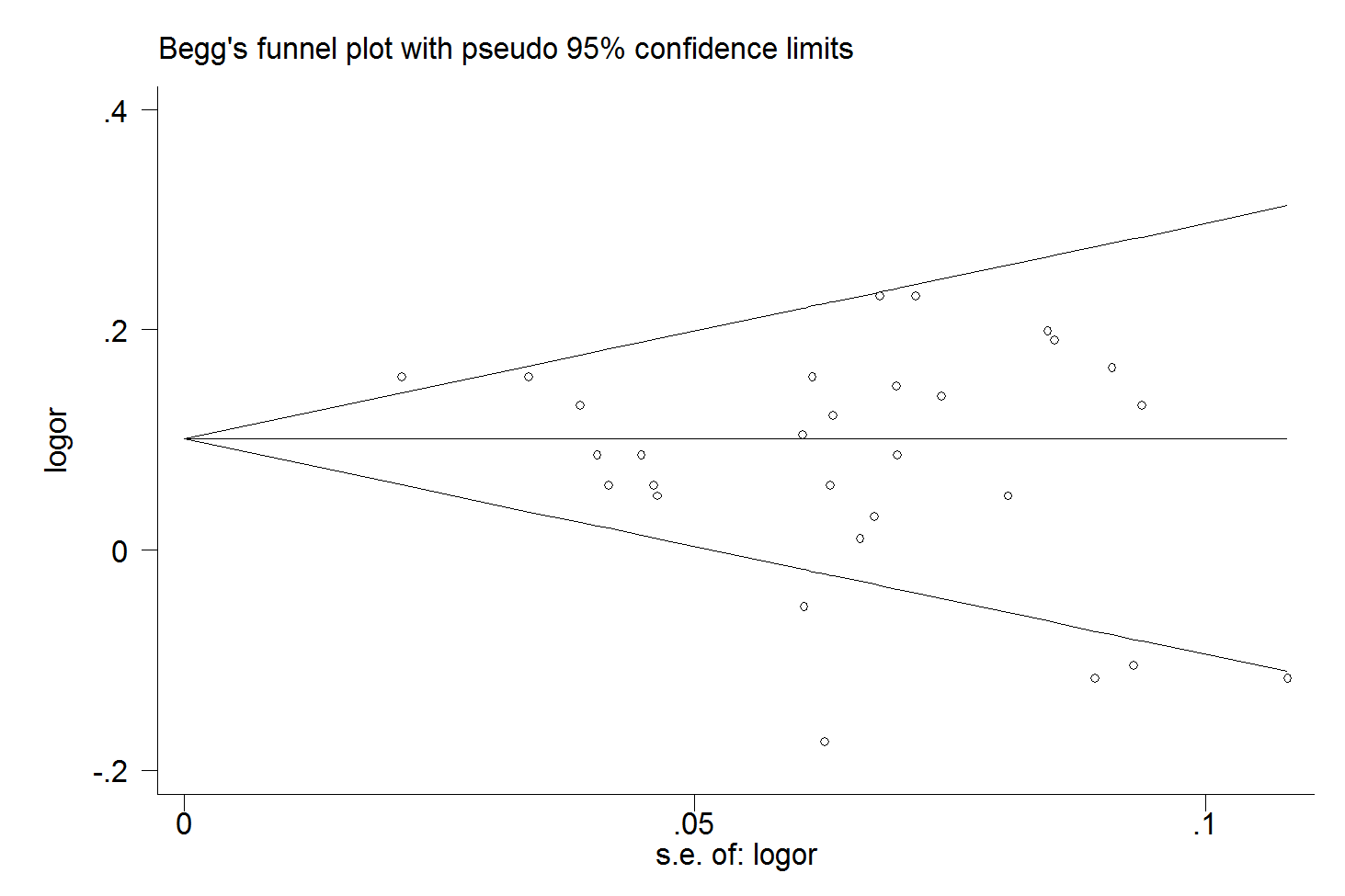

Supplement: Figure S4 — Begg's funnel plot of 5p12-rs10941679 polymorphism and BC risk. (TIF) [file pone.0073611.s004.tif]

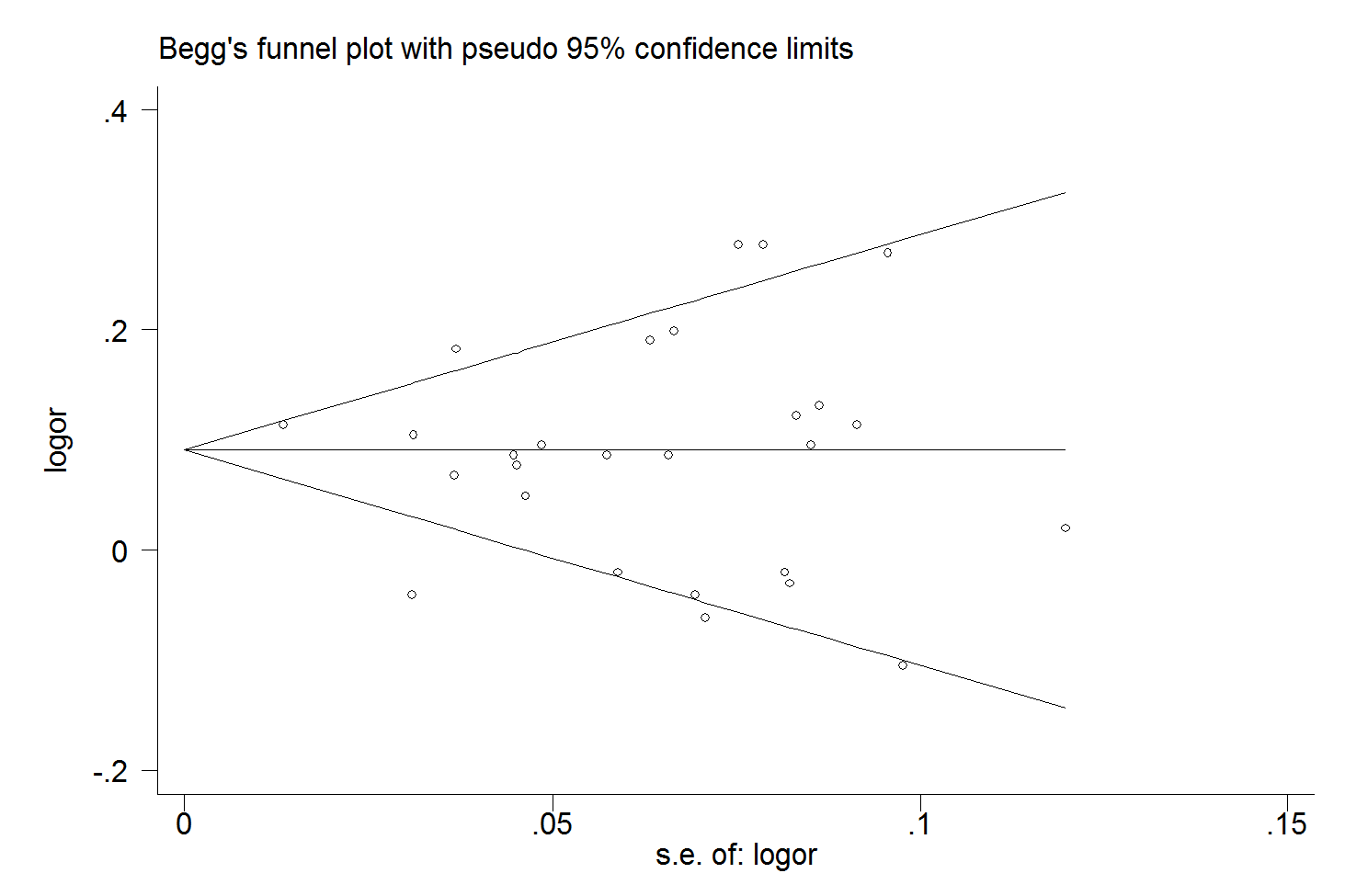

Supplement: Figure S5 — Begg's funnel plot of 5p12-rs4415084 polymorphism and BC risk. (TIF) [file pone.0073611.s005.tif]
